# Impact of Adjunctive VNS on Drug Load, Depression Severity, and Number of Neuromodulatory Maintenance Treatments

**DOI:** 10.3390/brainsci14020159

**Published:** 2024-02-04

**Authors:** Erhan Kavakbasi, Helen Bauermeister, Lars Lemcke, Bernhard T. Baune

**Affiliations:** 1 Department of Psychiatry, University Hospital Münster, University of Münster, 48149 Münster, Germanybernhard.baune@ukmuenster.de (B.T.B.); 2 Department of Neurosurgery, University Hospital Münster, University of Münster, 48149 Münster, Germany; 3 Department of Psychiatry, Melbourne Medical School, The University of Melbourne, Melbourne, VIC 3052, Australia; 4 The Florey Institute of Neuroscience and Mental Health, The University of Melbourne, Parkville, VIC 3052, Australia

**Keywords:** difficult-to-treat depression, treatment-resistant depression, esketamine, vagus nerve stimulation, electroconvulsive therapy, medication load

## Abstract

Vagus nerve stimulation (VNS) is a long-term adjunctive treatment option in patients with difficult-to-treat depression (DTD). A total of *n* = 20 patients (mean age 52.6 years) were included in the multicenter, prospective, observational, naturalistic RESTORE-LIFE study and were treated with adjunctive VNS as an add-on to treatment as usual. Exploratory and secondary outcome parameters from a single center were investigated for this present analysis. The overall mean drug load slightly decreased from 4.5 at baseline to 4.4 at 12 months (Z = −0.534, *p* = 0.594). The drug load was lower in previous electroconvulsive therapy (ECT) responders than in non-responders. There was a reduction in the mean number of hospitalizations per month after VNS implantation (Z = 1.975, *p* = 0.048) and a significant decrease in the mean Montgomery Åsberg Depression Rating Scale (MADRS) score from 27.3 at baseline to 15.3 at 12 months (T = 4.230, degree of freedom (df) = 19, *p* = 0.001). A history of ECT response at baseline was associated with greater improvement in the MADRS score after 12 months of VNS (F = 8.171, *p* = 0.013). The number of neuromodulatory maintenance treatments decreased during the follow-up period. In summary, there was an alleviation in the burden of illness among DTD patients treated with VNS.

## 1. Introduction

According to the World Health Organization (WHO), depression will be the greatest cause of the disease burden worldwide by 2030 [1]. Non-responsiveness to antidepressant treatment is a common phenomenon in the management of depression. The STAR*D trial revealed that the probability of an antidepressant response decreased with every unsuccessful treatment attempt, whereas the number of side effects and relapse rates increased after each trial. After two antidepressant trials, the probability of response to third-line treatments decreased to 13% [2]. Only about one-third of patients achieve stable remission after appropriate treatment; in all other cases, there is only a partial response without remission or even a non-response [3]. The term treatment-resistant depression (TRD) has mainly been used to describe patients who do not adequately respond to two antidepressant trials [4]. In this approach, depression is considered an acute disease with times of illness and remission [5]. Recently, the concept of difficult-to-treat depression (DTD) was suggested to describe cases in which remission is often not achievable and the disease is characterized by a chronic course with long-term reduced quality of life and functioning [6]. These patients fail to respond to multiple antidepressants and present residual symptoms and poor outcomes. The goal of treatment changes from complete remission to best symptom control and disease management to improve the quality of life and reduce suffering [6].

Electroconvulsive therapy (ECT) is one of the most important treatment options for patients with treatment-resistant depression and is strongly recommended by international guidelines [7]. The response rate to electroconvulsive therapy in TRD patients is about 58–70% [8]. After an acute series of ECT, maintenance sessions are often considered to reduce the risk of relapse. Esketamine and racemic ketamine are non-competitive antagonists of the N-methyl-d-aspartate (NMDA) receptor. In recent years, they have been clinically used in patients with treatment-resistant depression or suicidality [9]. They are typically administered intranasally or intravenously, leading to rapid relief of depressive symptoms and a reduction in suicidal ideation [10]. The antidepressant effect of esketamine is often short-lasting; thus, repeated administration has been suggested and practiced to prolong and increase its efficacy [10]. To preserve the improvement after the acute phase, the patients are offered a maintenance treatment [11]. Patients with DTD and a chronic course of the disease may require long-term complex antidepressant pharmacotherapy, as well as repeated maintenance treatments with ECT or esketamine. These interventions may be burdensome for patients because of their repetitive nature, raising the question of potential adjunctive treatment options to reduce the burden of the disease and treatment.

Invasive vagus nerve stimulation (VNS) is a long-term adjunctive treatment option for patients with unipolar or bipolar DTD who fail to achieve or sustain a response to standard treatment approaches. These patients usually require increased healthcare utilization due to frequent or prolonged hospital admissions [12]. Patients with a history of ECT response and need for maintenance sessions or relapse during maintenance treatment may be referred for VNS as an adjunctive treatment [12]. In a large registry, adjunctive VNS, in addition to treatment as usual (TAU), was superior to TAU alone in terms of a 5-year cumulative response rate (67.6% vs. 40.9%) [13]. VNS usually shows late-onset antidepressant efficacy of approximately 6–12 months. In the registry, the median time to first response was shorter in patients with VNS (12 months) than in those treated with TAU alone (48 months). Although patients with a history of ECT response seem to be more likely to benefit from adjunctive VNS, ECT non-responders for whom treatment options are limited have a higher response rate in the case of VNS + TAU than TAU alone [13]. In addition to improving depression symptoms, the possibility of reducing the amount and dose of medication, the number of hospitalizations, and the need for maintenance treatment sessions are some of the motivations of patients who consider VNS. However, little is known about the change in these disease and treatment-related factors during the long-term course of VNS treatment.

### Objectives and Research Questions

In this present article, we analyzed exploratory and secondary outcome data from a single center in the observational prospective naturalistic RESTORE-LIFE study [14]. We explored the change in treatment load for depression and illness burden following VNS. This analysis primarily focuses on the change in medication load from baseline to 12 months follow-up after VNS. We also investigated the need for additional treatments during the follow-up period. For the analysis of changes in disease severity, we investigated the change in the mean MADRS score from baseline to 12 months follow-up and analyzed if the ECT response status at baseline had an impact on the change in mean MADRS score. As another indicator of disease burden, we examined the mean number of hospitalizations after one year of VNS, comparing it to the mean number of admissions prior to VNS initiation.

## 2. Material and Methods

From October 2019 to July 2021, a total of *n* = 20 patients with unipolar or bipolar difficult-to-treat depression were included in the RESTORE-LIFE study and were implanted with an invasive VNS treatment system (Symmetry, LivaNova). The RESTORE-LIFE study is an international, multicenter, post-market observational, real-life study that evaluates the long-term outcomes of patients with DTD. The inclusion criteria of the study encompassed the diagnosis of bipolar or unipolar recurrent (>2 episodes) or chronic (>2 years) depression that failed to improve sufficiently after adequate antidepressant treatment steps according to local standards [14]. Patients with psychosis, mental retardation, severe substance use disorders, and severe personality disorders were excluded [14]. The primary endpoint of the study was response rate, defined as a >50% decrease in Montgomery Åsberg Depression Rating Scale (MADRS). A baseline assessment was performed 1–6 weeks prior to implantation after obtaining written informed consent. Institutional review board approval was obtained from the local Ethics Committee of the University of Münster (ClinicalTrials.gov Identifier: NCT03320304, https://clinicaltrials.gov/study/NCT03320304, accessed on 28 November 2023). This study was conducted in compliance with the ethical principles for medical research involving human subjects as stated in the Declaration of Helsinki.

The Mini International Neuropsychiatric Interview (MINI) was used to confirm primary psychiatric diagnosis and psychiatric comorbidities. Data on antidepressant treatment history, psychiatric medications, and neuromodulatory treatments were also collected. Postoperative evaluation and follow-up were performed every three months. In this present analysis, we provide single-center secondary and exploratory outcome data from the Department of Psychiatry, University Hospital Münster, Germany. This manuscript focuses on changes in medication load, number of hospitalizations, depression severity (mean change in MADRS), and neuromodulatory treatment with esketamine and ECT from baseline to 12 months after VNS implantation. After surgery, a two-week recovery period was recommended before the start of stimulation [15]. As in many other centers, we started dose titration of VNS treatment as soon as tolerated by the patients. On average, the first titration was performed 10 days after surgery (median, 7 days) during the outpatient visits. Output current was increased in steps of 0.25 mA until 1.5 mA was reached. The default VNS settings were as follows: ON time, 30 s; OFF time, 5 min; frequency, 20 Hz; and pulse width, 250 µs.

To determine medication load, an index was calculated for each psychiatric drug. The standard drug dose is assumed to be 1.0. This is the minimum dose of the drug to be considered as an adequate trial due to the antidepressant treatment history form. Compared to the standard dose, an index was determined for the actual dose of the drug. For example, tranylcypromine 10 mg was taken as 1.0, whereas 20 mg was taken as 2.0. For each patient, the indices of all the drugs were added to obtain the total drug load.

Statistical analyses were performed using IBM SPSS Statistics version 28.0.1.1. Descriptive statistics, as well as parametric and non-parametric tests, were conducted depending on the distribution of data to test for changes from baseline to 12 months. For parametric data, we used the *t*-test, and for non-parametric data, the Wilcoxon test (paired samples) or the Mann–Whitney U test (independent samples) was utilized. A multivariate model with repeated measures (Multivariate Analysis of Covariance, MANCOVA) was used to determine the impact of the previous ECT response status on the change in MADRS from baseline to 12 months. A *p*-value of <0.05 was considered statistically significant.

## 3. Results

### 3.1. Baseline Characteristics

A total of *n* = 20 patients were included in the analysis. The majority of patients (*n* = 14, 70%) were women. The mean age at implantation was 52.6 years. Patients were diagnosed with unipolar (*n* = 16, 80%) or bipolar (*n* = 4) major depression. Approximately half of the patients (*n* = 9, 45%) were diagnosed with at least one psychiatric comorbidity. Post-traumatic stress disorder (PTSD) was the most common psychiatric comorbidity. Interestingly, hypothyroidism was the most common comorbid medical condition, affecting more than half of the patients. The psychiatric history and disease characteristics revealed a high disease burden in our sample (Table 1). On average, the current depressive episode lasted for 28.4 months at the time of implantation. The mean age at onset of the first depressive episode was in young adulthood (30.0 years). The majority of patients (80%) have had five or more lifetime episodes of depression. The mean baseline MADRS score was 27.3. The baseline score on the MADRS item 10, rating suicidal ideation was 1.6 (median 1.5; SD, 1.5). The sample was characterized by a high degree of treatment-resistance. The mean number of failed antidepressant trials was 5.8 in the current episode and 12.2 in the previous episodes. Ninety percent (*n* = 18) of patients had a history of ECT treatment. Half of these patients were ECT responders (*n* = 9, 45.0%) and the other half were non-responders at baseline evaluation.

### 3.2. Adverse Effects of VNS Treatment

VNS therapy was usually well-tolerated. At 3 months, 40% of patients reported adverse events (40% hoarseness or voice alteration, 10% dyspnea during stimulation). At 6 months follow-up, the rate of adverse events decreased to 30%, which became 40% at 9 months (mostly hoarseness and voice alteration, one patient with pain during stimulation). At 12 months follow-up visit, 50% (*n* = 10) of patients reported stimulation-related adverse events (hoarseness, voice alteration 45%, pain 5%, dyspnea 5%). These adverse events did not lead to discontinuation of VNS treatment in any case.

### 3.3. Psychopharmacotherapy and Drug Load

We obtained retrospective data on medication load prior to VNS implantation. Due to retrospective data collection, there was variability in the time point of retrospective medication load assessment. On average, the reported retrospective medication load pertains to a time point 9.2 months (mean) prior to implantation (median 10.0). For instance, if VNS implantation was performed in June 2020, the retrospective assessment of medication load would pertain to September 2019, which is approximately 9 months prior to implantation. Prior to VNS, the patients received a mean of 2.9 different concomitant psychotropic agents. The mean number of drugs increased to 3.3 at baseline, the change was not statistically significant (Wilcoxon test, Z = −1.282, *p* = 0.200). The mean drug load prior to VNS was 4.0 (median 3.8, SD 2.4). The increase in drug load from prior to VNS to baseline (mean 4.5, SD 1.9) was not statistically significant (Wilcoxon test, Z = 1.255, *p* = 0.210). At baseline, the majority of patients (*n* = 17, 85.0%) were treated with three or more different agents. The mean number of psychopharmacological agents at baseline was 3.3 (median, 3.0; SD, 0.8), which decreased to 2.9 (median, 3.0; SD, 0.8) 12 months after implantation (Wilcoxon test, Z = 2.111, *p* = 0.035). Regarding drug load, one case was identified as an outlier using a box plot (9.7 at baseline, 15.8 at 12 months). This patient was excluded from the medication load analysis. The drug load at baseline (mean 4.5, median, 4.2; SD, 1.9) decreased to 4.4 (median, 4.0; SD, 2.0) at 12 months (Wilcoxon test, Z = −0.534, *p* = 0.594). In summary, the drug load increased from 9 months prior to VNS to baseline assessment. After initiation of VNS, there was a decrease in drug load from baseline to 12 months follow-up. However, these changes were not statistically significant. A summary of drug load is provided in Table 2.

**Figure 1:**
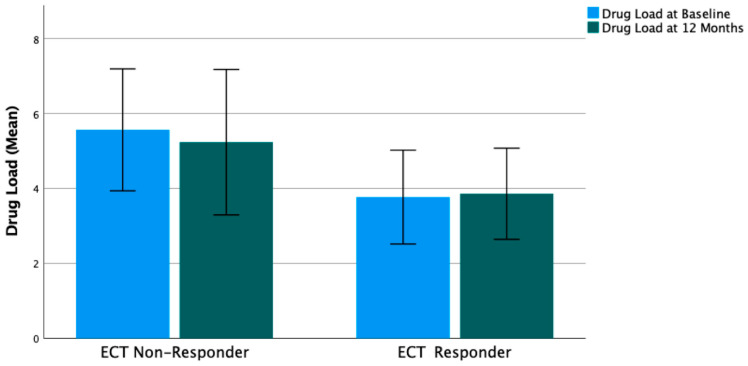
Patients with a history of previous ECT response had lower mean drug load at baseline (3.8 vs. 5.6, Z = −2.022, *p* = 0.046) and at 12 months (3.9 vs. 5.2, Z = −1.443, *p* = 0.167). Overall change of drug load from baseline to 12 months was not significant (Wilcoxon test, Z= −0.534, *p* = 0.594). Bars indicate 95% confidence intervals.

The drug load was lower in previous ECT responders than in ECT non-responders, both at baseline (3.8 vs. 5.6, Z = −2.022, *p* = 0.046) and at 12 months (3.9 vs. 5.2, Z = −1.443, *p* = 0.167) (Figure 1).

### 3.4. Hospitalizations

Hospitalizations were recorded during the baseline and follow-up visits. Short planned inpatient treatments necessary for the maintenance of ECT or esketamine without worsening of depression was not considered hospitalization. The mean number of lifetime psychiatric hospitalizations was 7.6 (median 6.5). In the last two years prior to VNS implantation, 80% (*n* = 16) of patients had at least one psychiatric hospitalization. The mean number of depression-related hospitalizations in the last two years was 2.25 (median 2.0), which corresponds to 0.094 hospitalizations per month (median 0.083) per case. One year after implantation, 15 hospitalizations were reported (0.75 per case). Approximately 45% (*n* = 9) of patients had at least one hospitalization in the 12 months after VNS implantation. Compared to two years prior to VNS implantation, there was a decrease in the mean number of hospitalizations per month to 0.0625 after VNS (median 0, Wilcoxon test, Z = 1.975, *p* = 0.048, Table 3).

### 3.5. Change in Depression Severity

**Figure 2:**
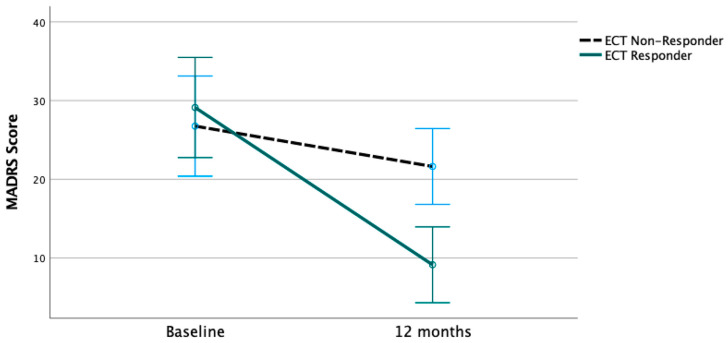
Change in MADRS Score depending on previous ECT response. ECT responders had stronger decrease in mean MADRS score from baseline to 12 months than ECT non-responders. Bars indicate 95% confidence intervals.

There was a significant decrease in MADRS scores from a mean of 27.3 at baseline to 15.3 at 12 months (T = 4.230, degree of freedom (df) = 19, *p* = 0.001). The baseline MADRS score was not significantly different between the ECT responders (mean MADRS 30.2) and non-responders (mean MADRS 26.0, Mann–Whitney U test, Z = 1.153, *p* = 0.258). However, at 12 months, there was a significant difference in MADRS scores between ECT non-responders (mean MADRS 21.4) and ECT responders (mean MADRS 9.3, Mann–Whitney U test, Z = −2.918, *p* = 0.002). Patients with a previous response to ECT showed a greater decrease in mean MADRS score from baseline to 12 months (Figure 2, Table 4). There was no significant difference in MADRS scores at baseline and 12 months between patients with and without hypothyroidism. In a multivariate model with repeated measures with time (MADRS score at baseline and MADRS at 12 months) as a dependent within-subject factor; ECT response status as an independent variable; and age, psychiatric comorbidity, and sex as covariates, there was a significant interaction of time × ECT response status (F = 8.171, *p* = 0.013). There was no time × within-subject factor interaction with the within-subject factors of age at the time of implantation (F = 0.011, *p* = 0.918), psychiatric comorbidity (F = 0.025, *p* = 0.877), or sex (F = 0.645, *p* = 0.436).

### 3.6. Maintenance Treatment with ECT or Esketamine

**Figure 3:**
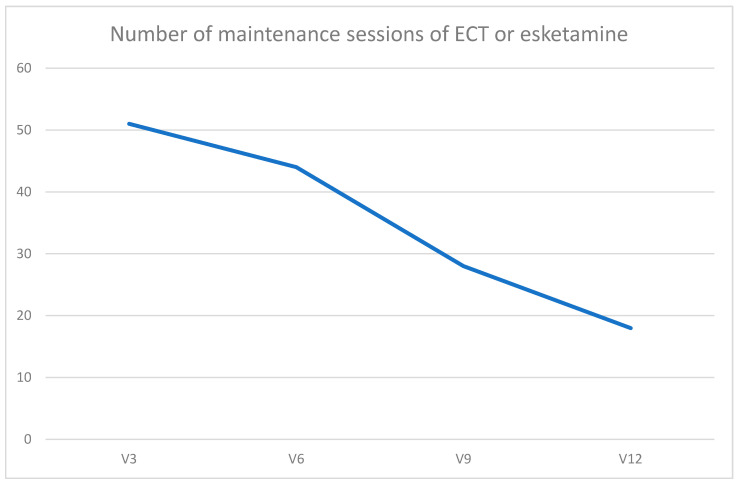
Number of maintenance treatment sessions in patients receiving either electroconvulsive therapy or esketamine maintenance treatment decreased from 3 months follow-up visit to 12 months follow-up visit. A clear decrease was seen after 6 months of VNS treatment.

During the follow-up period, a total of *n* = 9 patients received maintenance treatment either with ECT (*n* = 4) or esketamine (*n* = 5). The number of maintenance sessions was 51 at 3 months follow-up (between implantation and 3 months follow-up), 44 at six months, 28 at 9 months, and 18 at 12 months follow-up, respectively. Figure 3 illustrates this subsequent decrease in the number of maintenance treatment sessions during the follow-up period (Figure 3). The patients in the maintenance treatment regimen received a mean of 10.6 (SD 5.0) maintenance sessions of ECT or esketamine during the first 6 months after VNS implantation. In the second half of the observation period (months 7–12 after VNS implantation), the number of maintenance sessions per case in these patients decreased to a mean of 5.1 sessions (SD 3.1) per case. This decrease in the number of maintenance sessions from the first to the second half of the follow-up period was statistically significant (Wilcoxon test, Z= −2.530, *p* = 0.011), occurring after six months of VNS when the delayed onset of antidepressant efficacy of VNS was expected. Retrospective data on the number of maintenance sessions in the period 12 months prior to VNS implantation were available in *n* = 4 patients. In these patients, the mean number of maintenance sessions per case decreased from 14.8 (in the 12 months prior to VNS) to 13.5 in the 12 months after VNS implantation. However, this change was not statistically significant (Wilcoxon test, Z= −0.816, *p* = 0.414), probably due to the small number of cases.

Additionally to the patients included in the maintenance regimen, two other patients received one ECT series each without being included in a maintenance ECT regimen: one patient between baseline and 3 months (23 sessions), and another patient with a previous ECT non-response received a total of 20 ECT treatments between months 8 and 12. Furthermore, during the follow-up period, one other patient received an esketamine treatment series for the treatment of an acute crisis without being on a maintenance regimen.

## 4. Discussion

The psychiatric evaluation at baseline revealed a high disease burden in this sample. Almost half of the patients had at least one psychiatric comorbidity. A long duration of current depressive episode (28.4 months) as well as five or more lifetime episodes in most patients indicate a high burden of disease in this sample. Furthermore, a high number of failed antidepressant trials (5.8 current episode, 12.2 lifetime) as well as a high prevalence of previous ECT treatments underline a high degree of treatment resistance. These disease characteristics are in line with the recent report on baseline characteristics of the RESTORE-LIFE sample, revealing a high burden of disease [16]. The patient characteristics in our sample were also similar to those described in the 5-year registry; however, the baseline MADRS score was lower in the present analysis (27.3 vs. 33.1) [13]. The disease burden and resistance to treatment are typical characteristics of DTD. This term also refers to this sample and describes depression, which causes suffering despite adequate treatment [6]. VNS was considered in these patients to improve disease control and reduce the burden of the disease and treatment.

The possibility of reducing antidepressant medication over the long-term course of treatment is one of the motivations for patients to consider VNS as an adjunctive treatment. To date, there are no data on the impact of VNS on medication load in the long-term course in patients with DTD. We observed an increase in medication load from 9 months prior to VNS implantation to baseline assessment. In this present analysis, there was a significant reduction in the number of agents administered and a slight decrease in the mean medication load 12 months after initiation of VNS treatment (4.5 to 4.4). These results indicate that VNS may help reduce medication load in DTD patients. However, these changes were not statistically significant in our sample. This may be due to several reasons. Given the high burden of disease and the risk of relapse, clinicians and patients are cautious in terms of reducing medication. Furthermore, medication reduction was not the primary goal of this study or treatment, and the patients were treated in a naturalistic design. Given that the onset of antidepressant efficacy of VNS may occur with a latency of several months [17], it is reasonable to maintain medication for relapse prevention even in cases of improvement. Interestingly, patients with a history of ECT non-response had a higher medication load both at baseline and at 12 months, which can be explained by the strategy that in the case of ECT non-response treatment options are limited and healthcare professionals may tend to intensify medication more strongly than in ECT responders. As VNS is a long-term treatment, a clearer and more significant impact of VNS on medication load may occur after a longer follow-up period than the 12 months investigated in this analysis.

Regarding psychiatric hospitalizations, there was a significant decrease in the number of hospitalizations per month after 12 months of VNS compared to two years prior to implantation. Reduction in depression-related hospitalizations was also reported in a previous case series and may be one way that VNS reduces the burden of disease in DTD patients [18]. Despite the observed reduction in hospitalizations, almost half of the patients (45%) had at least one depression-related admission during the follow-up period, underlining the severity of the disease course in these patients.

This analysis revealed a significant decrease in the mean number of neuromodulatory maintenance sessions (ECT or esketamine) after six months of VNS treatment compared to the first six months of VNS. These results support the assumption of a delayed onset of the antidepressant action of VNS [17]. The number of maintenance sessions was higher in the first half of the observation period when VNS presumably did not exert full antidepressant efficacy. The antidepressant effect of VNS may result in a reduction of maintenance sessions after six months, whereas patients may need to continue frequent maintenance sessions in the first six months when an antidepressant effect of VNS is not yet expected. A similar impact of VNS on maintenance ECT treatment has previously been described in a case series of 10 patients. Seven of these patients discontinued maintenance ECT 12 months after VNS [18]. Our data support the assumption that VNS may reduce the need for maintenance neuromodulatory treatment sessions in both the ECT and esketamine maintenance regimens. In clinical practice, VNS can be offered to patients who require frequent maintenance sessions, which can be burdensome, to reduce the number of maintenance sessions. VNS could also be considered for patients who present with residual symptoms of depression despite maintenance treatments. Our results also support the strategy not to reduce maintenance sessions in the first six months of VNS.

Overall, there was a significant reduction in depression severity, which was stronger in patients with a history of an ECT response. There was a significant interaction effect of ECT response status at baseline on the improvement in the mean MADRS score from baseline to 12 months. These results are in line with those of other reports suggesting a better response to VNS in previous ECT responders than in non-responders [13]. However, ECT non-responders also experienced relevant symptom relief (MADRS 26.0 to 21.4). In the VNS registry, the cumulative response rate increased during the follow-up period of up to 60 months [13]. Thus, the reported favorable changes in depression severity, burden of disease, and treatment in our sample could be considered an early impact of VNS treatment after 12 months. Further improvement may occur during the subsequent years of VNS treatment. More than half of the patients were diagnosed with hypothyroidism, which was substituted with thyroid hormones. Previous meta-analyses have suggested a significant association between hypothyroidism and depression [19]. Preliminary data revealed a higher prevalence of hypothyroidism in patients with chronic and treatment-resistant depression than in those with non-TRD [20]. The high number of individuals with hypothyroidism in our sample may indicate a greater risk of treatment-resistant and chronic disease among patients with depression and comorbid hypothyroidism.

No serious adverse events related to stimulation were observed. However, at 12 months follow-up, 50% of patients reported stimulation-related typical adverse events such as hoarseness and voice alteration. Stimulation-related adverse events occur in up to two-thirds of patients with VNS, as reported previously [21]. Therefore, it is crucial to inform patients and caregivers of these adverse events before implantation. However, side effects are often well-tolerated and manageable. In our study, the side effects did not lead to discontinuation in any case.

## 5. Conclusions

In summary, after 12 months of VNS, there was a significant decrease in depression severity, particularly among previous ECT responders, as well as a reduction in the number of hospitalizations. The number of maintenance treatment sessions with ECT or esketamine decreased during the follow-up period. These results support the administration of VNS in the long-term treatment course of patients with DTD to reduce the burden of disease and treatment. The impact of VNS treatment may increase during the subsequent years of treatment beyond 12 months. VNS-related side effects were manageable and did not lead to discontinuation. A longer follow-up period may be necessary to observe more significant effects of VNS on the medication load. VNS is an important treatment option for patients with DTD to achieve optimal long-term disease management.

## 6. Strengths and Limitations

The strengths of this study include a prospective study design with regular follow-up visits and a follow-up period of 12 months. This study investigated a group of highly affected real-world individuals with difficult-to-treat depression and evaluated different treatment methods conducted in combination. This analysis addresses unique hypotheses and research questions, such as maintenance treatment and medication load in patients with VNS, which have not yet been answered. However, this was an observational naturalistic study. The lack of a control group, randomization, and blinding are the limitations of this study.

## Figures and Tables

**Table 1:** The baseline characteristics revealed high burden of disease in this sample.

Gender	w = 70 (*n* = 14)
	m = 30 (*n* = 6)
Age (years)	Mean 52.6
	Median 53.5
	SD 9.4
	Range 32–69
Diagnosis	
Unipolar MDD Bipolar Depression	80% (*n* = 16) 20% (*n* = 4)
Psychiatric Comorbidities	45% (*n* = 9)
Post-traumatic stress disorder Bulimia nervosa, dysthymia	25% (*n* = 5) Each 10% (*n* = 2)
Medical comorbidities	
Hypothyroidism Hypertension	55% (*n* = 11) 30% (*n* = 6)
Age of first onset of depression (years)	Mean 30.0
	Median 30.5
	Range 15–52
Duration of current depressive episode (months)	Mean 28.4
	Median 20.0
	SD 30.5
Number of failed antidepressant trials	
Current episode Previous episodes	Mean 5.8, median 5.0, SD 3.7 Mean 12.2, median 13.0, SD 6.3
Baseline disease severity	
MADRS	Mean 27.3, Median 27.5, SD 9.2

**Table 2:** We observed an increase in drug load from 9 months prior to VNS to baseline. The drug load subsequently decreased after 12 months of VNS treatment. However, the changes in drug load were not statistically significant.

Time Point	Mean Drug Load	Median Drug Load	*p*-Value for Change in Drug Load
Prior to VNS	4.0	3.8	*p* = 0.210 (pre-VNS-BL)
Baseline (BL)	4.5	4.2	
12 months follow-up	4.4	4.0	*p* = 0.594 (BL-V12)

**Table 3:** After VNS implantation, there was a significant decrease in the mean number of hospitalizations per year compared to the two years before VNS.

Number of Hospitalizations per Case and Year (Mean/Median)	Test Statistics (Wilcoxon Test)
Pre-VNS 1.125/1.0	Z = 1.975; *p* = 0.048
Post-VNS 0.75/0	

**Table 4:** Overall, the MADRS score significantly decreased from baseline to 12 months after VNS implantation (T = 4.230, *p* = 0.001). Patients with a history of previous ECT response showed stronger decrease in mean MADRS score, whereas baseline MADRS score was not significantly different between ECT responders and non-responders.

Mean MADRS (95% Confidence Interval)	Overall	ECT Responder	ECT Non-Responder	Test Statistics Mann–Whitney U Test
Baseline	27.3 (22.9–31.6)	30.2 (23.0–37.5)	26.0 (19.1–32.9)	Z = 1.153, *p* = 0.258
12 months	15.3 (11.4–19.3)	9.3 (4.0–14.6)	21.4 (16.6–26.3)	Z = −2.918, *p* = 0.002

## Data Availability

The data presented in this study are available on reasonable request from the corresponding author.
